# The Dietetic Treatment of Diabetes

**Published:** 1919

**Authors:** 


**The Dietetic Treatment of Diabetes.** By R. D. BASU, Major, I.M.S. (retired). Pp. 105. Seventh Edition. The Panini Office, Allahabad. 1916. Price Re. 1.8.

Major Basu's little work is highly interesting, and seven editions have appeared in as many years. He has had to treat diabetes among a vegetarian people, and his directions, in showing how this can be done, embody much sound common sense. Cocoa nut, dâls, ground nuts, unripe bananas, milk and certain preparations of rice, with green vegetables, some fruits and various oleaginous nuts and seeds afford a sufficient and varied diet. He avoids giving carbohydrates in the morning hours, but is apparently anxious to allow them and even wheaten bread later in the day. It is curious to note that either nut margarine or clarified butter (ghee) are safer than ordinary butter. He is strongly of opinion that diabetes is due to an alimentary toxæmia, but it is doubtful whether any of the theories of diabetes are capable of proof.

